# Diametric measurement of foveal avascular zone in healthy young adults using optical coherence tomography angiography

**DOI:** 10.1186/s40942-016-0053-8

**Published:** 2016-12-12

**Authors:** Nazimul Hussain, Anjli Hussain

**Affiliations:** 1 Department of Ophthalmology, Al Zahra Hospital, PO Box 3499, Sharjah, United Arab Emirates; 2 Al Buhairah Corniche, Sharjah, United Arab Emirates

**Keywords:** Optical coherence tomography angiography, Foveal avascular zone, Superficial capillary plexus, Deep capillary plexus, Horizontal diameter, Vertical diameter, Macula, Retinal vasculature

## Abstract

**Background:**

The objective of this study is to measure the diameter size of foveal avascular zone (FAZ) in both eyes of healthy young adults using optical coherence tomography (OCT) angiography.

**Methods:**

A cross sectional study to measure the foveal avascular zone in healthy young adults. Subjects underwent OCT angiography using Zeiss AngioPlex OCT angiography in both eyes.

**Results:**

30 eyes of 15 healthy young adults were evaluated. The mean horizontal FAZ diameter of superficial vascular plexus was 661.166 ± 119.99 microns and mean vertical FAZ diameter was 660.033 ± 96.169 microns (P = 0.9442). The mean horizontal FAZ diameter of deep vascular plexus was 1011.2 ± 154.526 microns and the mean vertical FAZ diameter of deep vascular plexus was 818.033 ± 102.108 microns. There was no difference between the contralateral eyes of subjects in FAZ diameter of both superficial and deep capillary plexuses.

**Conclusion:**

The mean diametric size of FAZ in superficial capillary plexus is 660.599 ± 0.801 microns and deep capillary plexus is 914.616 ± 136.589 microns. There was no significant difference with the contralateral eyes.

## Background

Optical coherence tomography angiography (OCT-A) is a new non-invasive imaging technique using motion control contrast imaging to high resolution volumetric blood flow information and generating a useful angiographic images without use of any injectable fluorescein dyes [1]. This new technology has opened a new dimension to understand and detect vascular abnormality in the various capillary plexuses in the retina. OCT-A has shown that capillary plexuses abnormality in the macula can be studied without the use of fluorescein dye.

OCT-A has also shown vascular density and foveal avascular zone (FAZ) abnormality in retinal vein occlusion, choroidal neovascularization and in eyes with diabetic retinopathy [2–8]. It has also helped to understand the middle capillary plexus abnormality in retinal vascular diseases [6]. Normal measurements of volume density of capillary plexus and foveal avascular zone have also been reported [4, 5, 8]. All these were measured in area (mm^2^) and predominantly in unilateral healthy eyes.

Several studies have shown the dimension of FAZ using fundus fluorescein angiography (FFA) and psychophysical test [9–11]. Wu et al. [9] have shown on FFA the mean longest diameter of FAZ measurement as 0.88 ± 0.16 mm, the horizontal diameter as 0.73 ± 0.15 mm and vertical diameter as 0.70 ± 0.17 mm. It has also been reported that the area of FAZ and the age of the subjects showed a statistically significant positive correlation (r = 0.383, P < 0.005). Psychophysical measurement of FAZ have also shown significant difference of FAZ size between younger age (average 24 years) and older age groups (average 61 years) [11].

With this background, we did a cross sectional study of diametric measurement of foveal avascular zone in superficial capillary plexus (SCP) and deep capillary plexus (DCP) in both eyes of healthy young adults.

## Methods

A cross sectional study was done during the period from May 2016 until June 2016 to measure the foveal avascular zone (FAZ) in healthy young adults. The participants belonged to ethnic race from Middle Eastern, Indian and Filipino. All underwent optical coherence tomography angiography (OCT-A) using Zeiss AngioPlex OCT angiography in both eyes. 6 × 6 mm OCT A Scans was used. After acquisition of the images, the superficial capillary plexus (SCP) FAZ diameter was measured both vertical and horizontal. Additionally, the deep capillary plexus (DCP) FAZ diameter was also measured similarly. SCP was defined as vascular layer detected in the scan at the level of inner retinal layer shown as superficial retinal layer in the En-face image acquired and DCP was defined as vascular layer detected in the scan at the level between inner nuclear layer and retinal pigment epithelium shown as deep retinal layer in the En-face image acquired. These images were acquired with inbuilt OCT Angiography software in the machine.

**Fig. 1:**
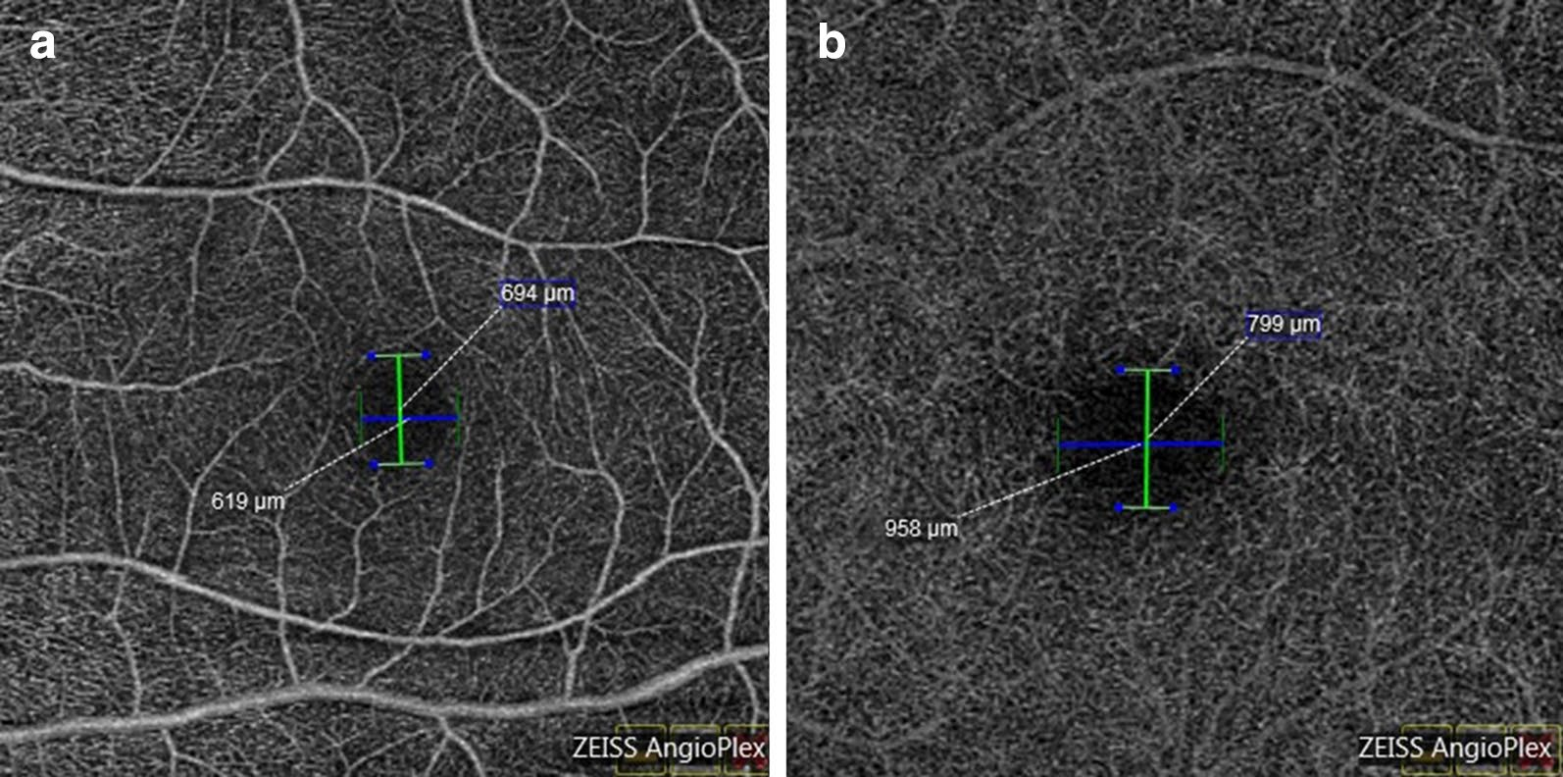
The calliper measurement of both horizontal and vertical diameters of superficial (**a**) and deep (**b**) capillary plexus

Measurement of SCP FAZ was done with the caliper provided in the Angioplex OCT. The diameter was measured from the inner most visible well defined vascular marking from one end to the other both horizontally and vertically (Fig. 1a). Similarly, the DCP FAZ diameter was measured from the inner most well defined visible vascular marking (Fig. 1b).

All the subjects had normal healthy eyes without any gross refractive error (<±0.5 D) and without any systemic diseases like diabetes, hypertension or pathology that may adversely affect the retinal vasculature. None of the subjects had any intervention in the eyes that may affect the measurements. Informed consent was taken from all the participants and study was approved by the institutional review board.

To test the significance of measurement of FAZ in both horizontal and vertical diameter of SCP and DCP and also between the contralateral eyes, Mann–Whitney U test was performed using 2 tail and with P value at <0.05.

## Results

**Fig. 2:**
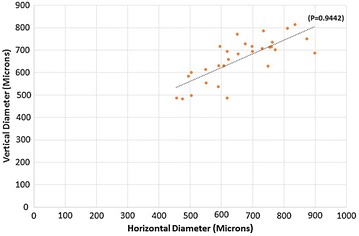
Scatter plot distribution of values of horizontal and vertical diameter of superficial capillary plexus

**Fig. 3:**
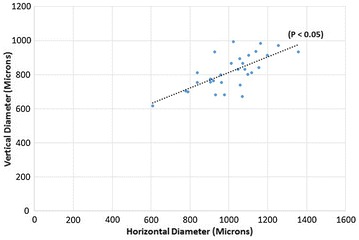
Scatter plot distribution of values of horizontal and vertical diameter values of deep capillary plexus

30 healthy eyes of 15 healthy young adults were evaluated. There were 6 males and 9 females with ratio of 2:3. The age ranged from 26 to 46 years with mean age of 36.07 ± 6.34 years (median 35 years). The mean horizontal FAZ diameter of superficial capillary plexus (SCP) was 661.166 ± 119.99 microns and mean vertical SCP FAZ diameter was 660.033 ± 96.169 microns. The difference between the horizontal and vertical SCP FAZ was not significant (P = 0.9442) (Fig. 2). The mean horizontal FAZ diameter of DCP was 1011.2 ± 154.526 microns and the mean vertical DCP FAZ diameter was 818.033 ± 102.108 microns. The difference between the vertical diameter and horizontal diameter in deep vascular plexus was significant (P ≤ 0.05) (Fig. 3).

Considering the mean vertical and horizontal diameter of SCP FAZ, the average diameter of SCP FAZ was calculated as 660.599 ± 0.801 microns. Extrapolating the radius of the mean diameter of SCP FAZ (r = 330.299 microns), the area of the SCP FAZ was estimated to be 0.34 mm^2^. Similarly, average diameter of the DCP FAZ was 914.616 ± 136.589 microns and the calculated area of DCP FAZ (r = 457.308 microns) was 0.66 mm^2^.

There was no difference between the contralateral eyes of each subjects in FAZ diameter of both superficial and deep capillary plexuses (horizontal SCP FAZ P = 0.9681; vertical SCP FAZ P = 0.6818; horizontal DCP FAZ P = 0.8025; vertical DCP FAZ P = 0.9681). However, there was significant difference between the SCP FAZ vertical and horizontal diameter with DCP FAZ horizontal and vertical diameter (P < 0.05) between the two eyes of same healthy subjects.

## Discussion

Several reports have shown that OCT-A is gradually becoming a valuable tool in imaging and quantifying the vascular abnormality in various retinal and choroidal vascular diseases [3–7].

Samara et al. [4] have shown the mean FAZ area in 17 eyes of branch retinal vein occlusion (BRVO) and 17 unaffected eyes. The mean superficial FAZ area was 0.312 mm^2^ in BRVO eyes and 0.284 mm^2^ in unaffected eyes (P = 0.54) whereas there was significant difference at the level of DCP between the BRVO and unaffected eyes (0.519 mm^2^ vs 0.410 mm^2^). This study found significant increase in the FAZ of DCP and not in the SCP. They have also shown that the volume density of vascular network was lower in the BRVO eyes compared to fellow eye in both networks.

Wons et al. [5] also compared the FAZ diameter in eyes with retinal vein occlusion(RVO) (n = 19 eyes) with the fellow healthy eyes (n = 19 eyes). It was reported that the FAZ diameter of superficial Vascular Layer was 707.07 ± 141.75 microns in healthy fellow eyes which was significantly different than in eyes with RVO (P = 0.008). In deep vascular layer, the FAZ diameter in healthy fellow eyes was 795.97 ± 144.85 microns which was significantly different in RVO eyes (P < 0.001). This study has also shown the enlargement of FAZ in both vascular layer in RVO compared to healthy fellow eyes as reported by Samara et al. [4].

Wang et al. [8] in the study of volume density in retina and choriocapillaris as measured by optical coherence tomography angiography in 105 Chinese eyes have shown the mean area of FAZ in superficial vascular network to be 0.35 ± 0.12 mm^2^. Shahlaee et al. [12] studied 34 eyes of 17 healthy subjects and reported the mean area of FAZ of SCP as 0.27 ± 0.10 mm^2^.

The present study differs from the reported literature, as it includes all healthy subjects and evaluates the FAZ diameter in both SCP and DCP with comparison to the fellow eyes. Our study reports the mean SCP FAZ diameter as 660.599 ± 0.801 microns and estimated mean area as 0.34 mm^2^. The findings are also similar to the area reported by Wang et al. [8] but the diametric measure of SCP FAZ was less than reported by Wons et al. [5]. The highlight of this study is inclusion of healthy eyes without any gross refractive error. It was shown by Wang et al. that refractive error affects the density of FAZ [8].

Our study estimated the mean DCP FAZ as 914.616 ± 136.589 microns and mean estimated DCP FAZ area as 0.66 mm^2^ which is larger than reported by Wons et al. [5]. This study particularly highlights that there is no difference between the contralateral eyes of each subjects in FAZ diameter of both superficial and deep capillary plexuses however there was difference between the mean SCP FAZ and DCP FAZ (P < 0.05).

Most of the studies done on FAZ measurements of SCP and DCP used the RetiVue 100 XR Avanti as shown in Table 1 [1, 4, 5, 8, 12, 14] except Kuehlewein et al. [13] performed OCT Angiography with prototype swept source laser OCT (510 K clearance pending) from Carl Zeiss Meditec (Dublin, CA, USA). We used commercially available Zeiss Angioplex Cirrus HD OCT. Table 1 also highlights the consistent larger size of DCP FAZ in all the studies mentioned [4, 5, 8, 12–14]. Hence, the physio-anatomical characteristics of DCP FAZ in healthy eyes suggest the capillary perfusion of DCP. This has been clearly shown in most of the studies of OCT angiography in retinal vein occlusion and diabetic retinopathy where the DCP FAZ is affected in greater proportion than the SCP FAZ [4–8, 12, 15, 16]. This also suggest that DCP is susceptible to greater damage than SCP in retinal vascular disease or any ischemic insult on the macula.

**Table 1:** OCT machine and size of FAZ measured by different studies in healthy eyes

Study (n = eyes)	Type of OCT/technique	Mean size of FAZ
Samara et al. [4] (n = 17)	Avanti SD OCT (RTVue-XR Avanti; Optovue)	SCP: 0.284 (0.206–0.362) mm^2^ DCP: 0.410 (0.304–0.517) mm^2^
Wons et al. [5] (n = 19)	RTVue 100 XR Avanti (Optovue)	SVL: 707.07 ± 141.75 µm DVL: 797.97 ± 144.85 µm
Wang et al.[8] (n = 105)	RTVue 100 XR Avanti (Optovue)	SRVL: 0.35 ± 0.12 mm^2^ DRVL: Not available
Shahlaee et al. [9] (n = 34)	RTVue 100 XR Avanti (Optovue)	SVL Horizontal: 0.59 ± 0.126 mm Vertical: 0.56 ± 0.118 mm DVL Horizontal: 0.69 ± 0.123 mm Vertical: 0.63 ± 0.110 mm
Kuehlewein et al. [10] (n = 19)	Prototype SS laser OCT (Carl Zeiss Meditec, Dublin CA, USA)	SRL: 0.304 ± 0.132 mm^2^ DRL: 0.486 ± 0.162 mm^2^
Yu et al. [11] (n = 76)	RTVue-XR Avanti (Software Version 2.0.5.39; Optovue)	SVL CFZ: 0.42 mm^2^ (male) 0.52 mm^2^ (female)
Present study Hussain and Hussain (n = 30)	Zeiss Angioplex (Cirrus HD OCT)	SCP Horizontal: 661.166 ± 119.99 µm Vertical: 660.033 ± 96.169 µm DCP Horizontal: 1011.2 ± 154.526 µm Vertical: 818.033 ± 102.108 µm

*SCP* superficial capillary plexus, *DCP* deep capillary plexus, *SVL* superficial vascular layer, *DVL* deep vascular layer, *SRVL* superficial retinal vascular layer, *DRVL* deep retinal vascular layer

The study limitation is the limited sample size. However, a larger sample size would add strong normative data in these ethnic race. This study attempted to provide a normative data as it tried to describe a finding and not a phenomenon, establish a standard, created a platform for further research and cross sectional study with participants from almost similar ethnicity with pigmented fundi.

However, it has few advantages. Firstly, it compared the measurement with the contralateral eye and Secondly, included healthy young participants from middle eastern, Indian and Filipino ethnic race with pigmented fundi. Thirdly, study participants had low refractive error (±0.5 D).

## Conclusion

OCT-A is a useful imaging technology to evaluate the vascularity of superficial and deep capillary plexus. Normative data will contribute to the average change in the FAZ diameter in the retinal vascular diseases. This study has shown that there is no difference in the FAZ diameter of SCP and DCP between the contralateral eyes of healthy young adults. Based on this study, future development of diametric mapping of FAZ to the already available software of area mapping is another importance of this study which can be of clinical relevance in follow up of retinal vascular disease affecting the macula.
